# Editorial: Osteoporosis in Rheumatic Diseases, What's New?

**DOI:** 10.3389/fmed.2021.808345

**Published:** 2021-12-03

**Authors:** Barbara Ruaro, Serena Guiducci, José A. P. da Silva, Barbara Wade, Elisa Baratella, Marco Confalonieri

**Affiliations:** ^1^Pulmonology Department, University Hospital of Cattinara, University of Trieste, Trieste, Italy; ^2^Division of Rheumatology, Department of Experimental and Clinical Medicine, University of Firenze, Florence, Italy; ^3^Rheumatology Department, Centro Hospitalar e Universitário de Coimbra, Coimbra, Portugal; ^4^Faculty of Medicine, University of Coimbra, Coimbra, Portugal; ^5^Azienda Ospedaliero Universitaria (AOU) City of Health and Science of Turin, Department of Science of Public Health and Pediatrics, University of Torino, Torino, Italy; ^6^Department of Radiology, Department of Medicine, Surgery and Health Science, University of Trieste, Trieste, Italy

**Keywords:** osteoporosis, rheumatic diseases, connective tissue diseases, fragility fracture, bone mineral density (BMD), trabecular bone score (TBS), systemic sclerosis, rheumatoid arthritis

This Research Topic collection entitled “*Osteoporosis in Rheumatic Diseases, What's New?”*, compiled by authors from various countries, confirms that patients with rheumatic diseases are frequently also affected by osteoporosis (OP). All the articles included reported that OP is a major comorbidity in rheumatic patients due to factors like, chronic treatment, inflammation, immobility, low body mass index (BMI), and low vitamin D serum concentrations.

In the first article of the collection, the authors report on the effects vitamin D supplementation has on patients affected by both rheumatoid arthritis (RA) and OP. Interestingly, they observed that RA patients with OP on bisphosphonates and vitamin D supplementation had a higher BMD increase than those who did not receive the supplementation (Kwon et al.). They also emphasize that a vitamin D supplementation dose of ≥1,000 IU/day is more efficacious than one of 800 IU/day.

The second article underlines the role a surgeon's ability plays to evaluate all the parameters to improve the treatment of patients with advance knee OA (Molfetta et al.). The authors report that these patients have high risks of fragility fractures, e.g., vertebral and non-vertebral, e.g., the hip making it a must to obtain detailed information on bone mineral density (BMD). They also stress that more attention should be paid to identify and treat OP in elderly female patients with advanced knee OA with a total knee prosthesis (TKP). The third article focuses on some new imaging techniques, such as high-resolution peripheral quantitative computed tomography and trabecular bone score (TBS), for the evaluation of bone microarchitecture in patients with Axial Spondyloarthritis (axSpA) (Lim and Kang). Indeed, the authors report that BMD assessment by dual-energy X-ray absorptiometry, which is the primary diagnostic method for OP, may lead to an overestimation of bone density in axSpA as these patients often have abnormal calcification of spinal ligaments or syndesmophytes. This is in agreement with the fourth article where the authors argue that BMD may not provide adequate information on bone microarchitecture, and propose TBS be used as a diagnostic tool for the assessment of bone quality (Ruaro et al.). They demonstrate that systemic lupus erythematosus (SLE) patients have a higher frequency of trabecular bone loss and an increased risk of lower bone mass than healthy subjects. In the fifth article the authors discuss the utility of the Bone Strain Index (BSI), which includes local information on density distribution, bone geometry and loading (Ulivieri et al.). BSI differs from BMD and other variables of bone quality, including TBS, as they are all based on the quantification of bone mass and distribution averaged over the scanned region. In conclusion, the authors observe that BSI appears to be a powerful tool to assess bone strength and provides information on the physics of the skeleton resistance. In their narrative review, the authors of sixth manuscript, highlight that patients affected by rheumatic diseases run a high risk of low bone mass, evaluated by BMD, adding that TBS, which provides information on the bone microstructure, is also lower in these patients than in healthy subjects (Ruaro et al.). The authors of seventh article explore the relationship between different biologic mediators involved in the development of osteoporosis in RA patients (Llorente et al.). They underline that the RANK/RANKL/OPG and the Wnt/DKK-1/sclerostin pathways play a crucial role not only in the development of systemic and local OP, but also in bone erosions. Furthermore, different pro-inflammatory cytokines, i.e., TNF-α, IL-6, and IL-17 and autoantibodies, i.e., ACPA and anti-CarPA, play a relevant role in bone homeostasis regulation. They emphasize that biological therapies allow for the reversal of some of the negative effects RA has on bone and that they even seem to reduce the risk of osteoporotic fractures. The authors of the eighth article used dual X-ray absorptiometry (DXA) and the skeletal muscle mass index (RSMI) to make a prospective comparison of the long-term efficacy of bisphosphonates vs. denosumab, in a cohort of 98 elderly patients at 1 year from hip surgery (Pizzonia et al.). There was a slight, non-significant osteo-metabolic improvement in the alendronate group compared to the denosumab group and, interestingly, the denosumab group had a positive trend in the RSMI index.

In another review the researchers report that OP is a hallmark of inflammatory arthritidies and underline that, under these conditions, OP pathogenesis is, mainly driven by the predominant inflammation (Rotta et al.). This observation is an important feature in the evaluation of novel therapeutic agents for the treatment of inflammatory arthritidies. The pivotal role adequately controlling the inflammatory process plays was also confirmed in the tenth study where 128 patients with early rheumatoid arthritis (ERA) were enrolled (Bruno et al.). The authors observed that the presence of bone erosions is associated with systemic bone loss as from the earliest RA phases, suggesting that the inflammatory burden and autoimmune biology, that underpin RA, represent crucial enhancers of bone remodeling both at local and systemic levels.

The eleventh article carried out a bibliometric study summarizing the trends and developments in the field of osteoporosis in RA (Wu et al.). The study emphasizes that RA should be classified not only as a chronic disabling arthritis, but also as a condition leading to reduced bone mass. Indeed, they emphasize that multiple studies have demonstrated that RA patients frequently develop osteoporosis which may well lead to a reduced quality of life and an increase in healthcare costs.

In conclusion, this collection of papers stresses the importance of assessing osteoporosis in patients affected by rheumatic diseases by the use of a combination of standard methods, e.g., DXA and new techniques, e.g., the TBS score, with the aim of achieving an optimal management of this frequent and serious complication. Furthermore, numerous studies have emphasized the importance of vitamin D supplementation and the use of antiresorptive therapies, such as alendronate and denousumab, to enhance not only treatment regimens, but also the patients' quality of life. Last but not least, all the studies underline the pivotal role correctly treating rheumatic diseases plays in the prevention of secondary pathologies, such as osteoporosis.

## Author Contributions

BR, SG, and JS conducted the manuscript. EB and MC made the final amendments and approved the final version. All authors contributed to the article and approved the submitted version.

## Conflict of Interest

The authors declare that the research was conducted in the absence of any commercial or financial relationships that could be construed as a potential conflict of interest.

## Publisher's Note

All claims expressed in this article are solely those of the authors and do not necessarily represent those of their affiliated organizations, or those of the publisher, the editors and the reviewers. Any product that may be evaluated in this article, or claim that may be made by its manufacturer, is not guaranteed or endorsed by the publisher.

